# Factors influencing lysis time stochasticity in bacteriophage λ

**DOI:** 10.1186/1471-2180-11-174

**Published:** 2011-08-02

**Authors:** John J Dennehy, Ing-Nang Wang

**Affiliations:** 1 Department of Biological Sciences, University at Albany, 1400 Washington Avenue, Albany, NY 12222, USA; 2 Biology Department, Queens College, and the Graduate Center of the City University of New York, Flushing, NY 11367, USA

## Abstract

**Background:**

Despite identical genotypes and seemingly uniform environments, stochastic gene expression and other dynamic intracellular processes can produce considerable phenotypic diversity within clonal microbes. One trait that provides a good model to explore the molecular basis of stochastic variation is the timing of host lysis by bacteriophage (phage).

**Results:**

Individual lysis events of thermally-inducible λ lysogens were observed using a temperature-controlled perfusion chamber mounted on an inverted microscope. Both mean lysis time (MLT) and its associated standard deviation (SD) were estimated. Using the SD as a measure of lysis time stochasticity, we showed that lysogenic cells in controlled environments varied widely in lysis times, and that the level of lysis time stochasticity depended on allelic variation in the holin sequence, late promoter (*p*_*R*_*'*) activity, and host growth rate. In general, the MLT was positively correlated with the SD. Both lower *p*_*R*_*' *activities and lower host growth rates resulted in larger SDs. Results from premature lysis, induced by adding KCN at different time points after lysogen induction, showed a negative correlation between the timing of KCN addition and lysis time stochasticity.

**Conclusions:**

Taken together with results published by others, we conclude that a large fraction of λ lysis time stochasticity is the result of random events following the expression and diffusion of the holin protein. Consequently, factors influencing the timing of reaching critical holin concentrations in the cell membrane, such as holin production rate, strongly influence the mean lysis time and the lysis time stochasticity.

## Background

Some phenotypic variation arises from randomness in cellular processes despite identical environments and genotypes [1-9]. Population heterogeneity, resulting from such molecular stochasticity, has been documented in many microbial organisms including bacteriophage (phage) λ [10-13], *Escherichia coli *[14-16], *Bacillus subtilis *[17,18] and *Saccharomyces cerevisiae *[19-24]. This within-population variation can have far reaching life history consequences. For example, experimentally reducing noise in the expression of ComK decreased the number of competent *B. subtilis *cells in one study [18]. In another study, mutants of *S. cerevisiae *showing greater heterogeneity in survival had higher rates of occasional-cell survival during high stress conditions than did wild-type cells [25].

Because of their simplicity and ease of manipulation, phages are excellent models to explore the life history consequences of molecular stochasticity. Many phages use a "holin-endolysin" system to compromise two physical barriers, the cell membrane and the peptidoglycan layer, in order to lyse an infected host cell [26,27]. Although there are some variations on the theme, holin usually forms a hole(s) in the inner membrane, thus either allowing soluble endolysin into the periplasmic space [28,29] or activating the membrane-tethered endolysin already translocated to the periplasm [30-32]. Endolysin then digests the peptidoglycan, causing host cell lysis.

The most extensively studied lysis system is that of phage l, which consists of four genes: *S *(encodes holin and antiholin), *R *(encodes endolysin), *Rz*, and *Rz1 *(encode an integral inner membrane protein and an outer membrane lipoprotein, respectively). All genes are co-transcribed from the late promoter *p*_*R*_*' *during the late phase of the lytic cycle [26,27,33,34]. Under typical laboratory conditions, only *S *and *R *are needed for host lysis, though both *Rz *and *Rz1 *are essential in the presence of high concentrations of divalent cations [33-35].

The lytic pathway of phage λ is commonly divided into the early, delayed early, and late phases. Transitions between stages are triggered by well-characterized molecular actions involving gene transcription and translation [36]. Consequently, the timing of when individual cells enter each phase greatly influences the length of individual lysis times. A recent study by Amir *et al. *[10] showed that 69% of the total lysis time variance is due to variation in the time interval between the onset of the *p*_*R*_*' *promoter and the eventual lysis (see APPENDIX A). This observation suggests that a large portion of the observed lysis time stochasticity is a *de novo *phenomenon, confined to the production and accumulation of holin proteins in the cell membrane, rather than a direct carryover from the various upstream stochastic events.

The formation of the λ holin hole in the membrane is hypothesized to be a multi-step process that starts with the transcription of the late mRNA and the translation of the S holin protein. The resulting holin monomers are then inserted into the cell membrane, where they dimerize, then oligomerize [37], eventually leading to the formation of higher-order holin aggregates, or rafts, in the cell membrane. At a time that is specific to the holin protein sequence, the holin rafts are transformed into a membrane lesion(s) > 300 nm across [38], which is large enough for the passage of a 500 KDa protein [28,29]. Lysis ensues after endolysin digests the peptidoglycan. Thus, by regulating endolysin's access to the peptidoglycan, holin controls the timing of lysis [26,27].

**Figure 1:**
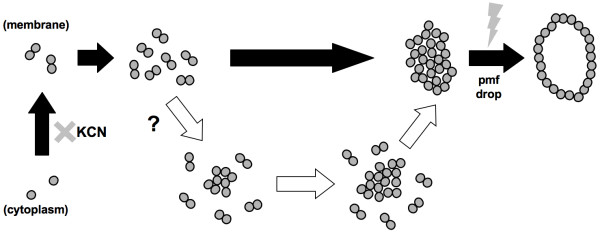
**Schematic presentation of two models of holin hole formation**. Holin monomers (shaded circles) are produced in the cytoplasm, and then transported to the cell membrane (a top-down view of the cell membrane thereafter) where they dimerize. A previous model (open arrows) [28,39] hypothesized that the growth of the holin aggregates ("rafts") to a critical size that is responsible for the collapse of the proton motive force (pmf), thus resulting in hole formation. The current model [40] (filled arrows) suggests that the holin proteins are mostly in a mobile state, then quite suddenly, aggregates are formed, leading to the formation of holin holes. Addition of the energy poison KCN halts further holin production and abolishes the pmf. This figure is adapted from Wang *et al. *[28] and White *et al. *[40].

To formalize the heuristic model of holin hole formation described by Wang *et al. *[28], Ryan and Rutenberg [39] proposed a two-stage nucleation model, in which the production rate of the holin monomers and holin self-affinity contribute to the aggregation of holin rafts. Raft aggregation is opposed by thermal Brownian motion which tends to disintegrate rafts into their holin constituents. As the rafts grow and then exceed a certain critical size (the first stage of nucleation), the probability of a second stage nucleation (triggering to hole formation) increases (Figure 1). According to this model, lysis time stochasticity is the inevitable outcome of each infected cell in the population following its own time course of growth in holin raft size. However, a recent study [40] using C-terminus GFP-fused λ S holin protein showed that, for most of the latent period, holin proteins are distributed uniformly in a relatively mobile state in the cell membrane. At a time that coincided with the triggering time, large immobile holin rafts suddenly appeared in the membrane. The transition from uniformly distributed holin to holin rafts occurred in less than a minute. Although it is not clear whether these large rafts correspond to the membrane holes observed by cryoelectron microscopy [38], this study nevertheless casts doubt on the previously hypothesized importance of holin raft size growth as the determining factor in lysis timing [28,39]. Rather, it is proposed that the lysis time is determined by when a critical holin concentration is reached in the cell membrane (Figure 1). According to this model, lysis time stochasticity is mainly the result of variation in the timing of reaching the critical holin concentration in the membrane.

Typically, the lysis time of a phage is estimated using a one-step growth curve [41-43]. In the case of phage λ, however, the availability of thermally-inducible *E. coli *λ lysogens allows a more precise determination of the lysis time by following the decline of culture turbidity [26,44]. Direct observation of the lysis of individual λ lysogenic cells [45] confirmed that the precipitous decline of culture turbidity, commonly observed among thermally-induced λ lysogen cultures, is a reflection of the saltatory nature of individual lysis events at the microscopic level. However, it is not clear to what extent the seemingly high synchronicity of lysis is influenced by various aspects of phage biology and host growth conditions. In this study, we used a simple experimental setup to assess how lysis time stochasticity is affected by allelic variation in the S protein, late promoter *p*_*R*_*' *activity, host growth rate, and the timing of energy poison KCN addition. Our results establish the ranges and limits of lysis time stochasticity under various conditions.

## Results

**Figure 2:**
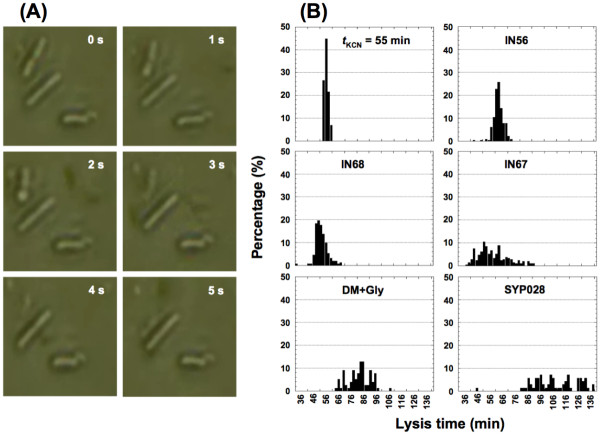
**Samples of a lysis recording and frequency distributions of various experimental treatments**. (A) Sample recordings from strain IN63. It takes about 5 sec for the upper left cell to disappear from view. (B) Sample frequency distributions of lysis times from strains IN56, IN67, IN68, SYP028, IN56 with KCN added at 55 min after thermal induction, and IN56 grown in glycerol minimal salts medium. The bin size was 2 min. Additional data are shown in Tables 1 and 2.

Using a microscope-mounted, temperature-controlled perfusion chamber, we observed and recorded individual lysis events of thermally-induced *Escherichia coli *l lysogens (Figure 2A). These observations revealed a considerable amount of variation in lysis time for the wild-type (WT) λ phage (Table 1; Figure 2B). Although the mean lysis time for the WT λ phage was 65.1 min, lysis times for individual lysogenic cells ranged from 45.4 to 74.5 min. Given that phage progeny accumulate linearly at ~7.7 phage per minute beginning ~28 min after lysis induction [46], the ~30 min range of lysis times could result in a three-fold difference in burst size between phages that lyse early and those that lyse late. This result motivated further exploration of variation in lysis time among other λ strains.

**Table 1:** Effects of holin allelic sequences on the stochasticity of lysis time.

Strain	***n***^***a***^	MLT (min)	SD (min)
IN61	274	45.7	2.92
IN56 (WT)	230	65.1	3.24
IN160	47	29.5	3.28
IN62	136	54.3	3.42
IN70	52	54.5	3.86
IN57	53	47.0	4.25
IN69	119	45.0	4.38
IN63	209	41.2	4.55
IN64	63	48.4	4.60
IN68	153	54.1	5.14
IN66	189	82.2	5.87
IN67	212	57.6	6.71
IN65	33	83.8	6.95
IN71	49	68.8	7.67

^*a *^In some cases, the sample size *n *is the pooled number of cells observed across several days. Detailed information can be found in Table S1 of additional file 1.

### Effect of allelic variation in holin sequence

It has long been known that different holin alleles show different lysis times [37,46,47]. However, it is not clear to what extent allelic differences in holin protein would affect the lysis timing of individual cells. To gain further insight, we determined the MLTs (mean lysis times) and SDs (standard deviations) of lysis time for 14 isogenic l lysogens differing in their *S *holin sequences (see APPENDIX B for our rationale for using SD as the measure for lysis time stochasticity). The directly observed MLTs (Table 1) were longer than those reported previously [46]. This discrepancy was mainly due to the fact that, in previous work, lysis time was defined by the time point when the turbidity of the lysogen culture began to decline, whereas in our current measurement, it was the mean of all individual lysis times observed for a particular phage strain.

**Figure 3:**
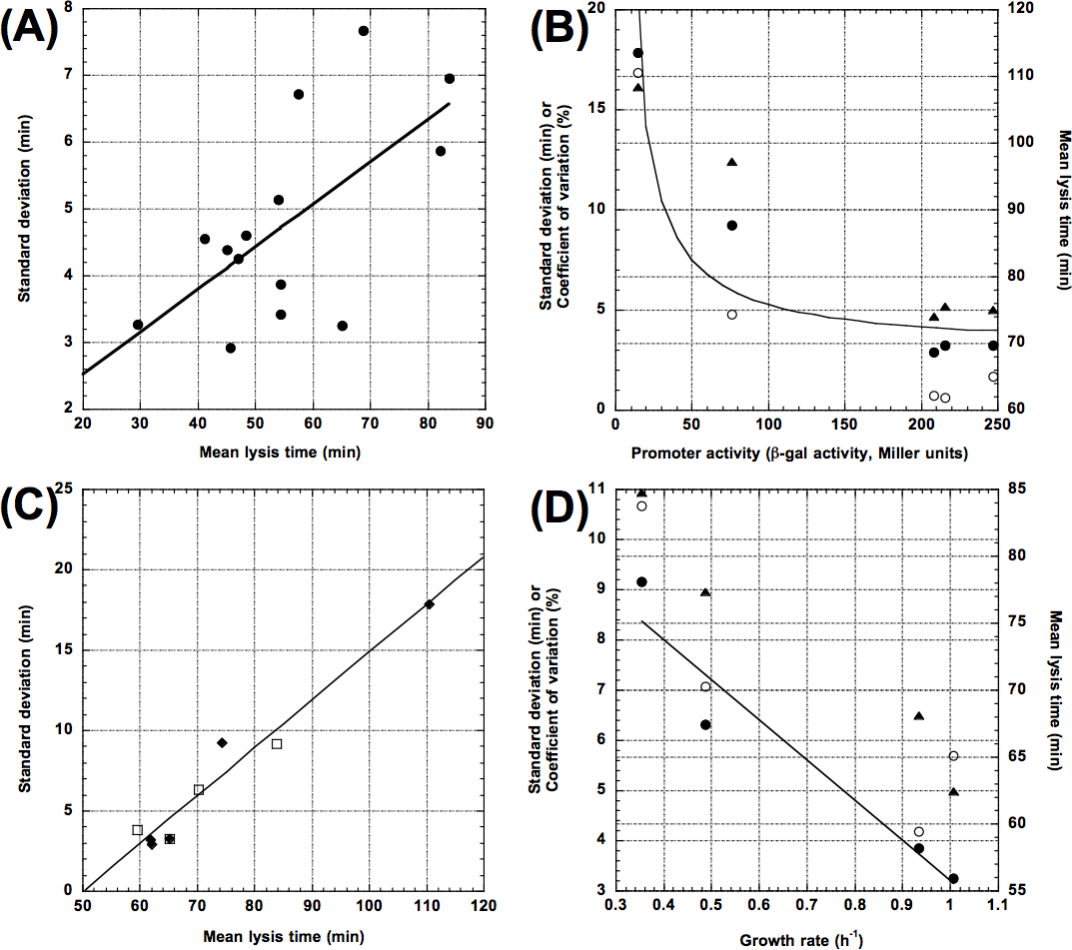
**Factors influencing λ lysis time stochasticity**. (A) Effect of allelic variation in holin proteins on mean lysis times (MLTs) and standard deviations (SDs). (B) Effect of λ's late promoter *p*_*R*_*' *activity [50] on MLTs, SDs and CVs (coefficients of variation). Solid curve is *SD *= 3.05 (72.73 + *P*)/*P*, where *P *was the *p*_*R*_*' *activity. (C) Effects of *p*_*R*_*' *activity and host growth rate on lysis time stochasticity. The regression line was obtained by fitting all data points from the late promoter activity (filled diamonds) and lysogen growth rate (open squares) treatments, except for the datum with the longest MLT and largest SD (from SYP028 in Table 2). (D) Effect of lysogen growth rate on MLT, SD, and CV. The fitted solid line shows the relationship between the growth rate and SD. All data are from Tables 1 and 2. Symbols: open circles, MLT; close circles, SD; closed triangles, CV.

Figure 3A revealed a significant positive relationship between MLT and SD (*F*_[1,12]_ = 8.42, *p *= 0.0133). However, we did not observe a significant relationship between MLT and another commonly used measure of stochasticity, the coefficient of variation (CV, defined as SD/MLT; [15,25,48,49]) (*F*_[1,12]_ = 1.50, *p *= 0.2445), indicating a proportional increase of the SD with the MLT. Figure 3A also reveals a relatively scattered relationship between the MLTs and the SDs (adjusted *R*^2 ^= 0.363), with several instances in which strains with similar MLTs are accompanied by very different SDs. For example, the mean lysis times for IN56 and IN71 were 65.1 and 68.8 min, but the SDs were 3.2 and 7.7 min, respectively. Apparently the observed positive relationship is only a general trend, not an absolute. The scattering of the plot also suggests that different missense mutations in the holin sequence can influence MLT and SD somewhat independently.

### Effect of late promoter *p*_*R*_*' *activity

Transcription of the late genes, including the holin gene *S*, from the *p*_*R*_*' *promoter marks the beginning of the late stage of the λ lytic development [36]. Since the major determinant of lysis time is thought to be when a critical holin concentration is reached in the cell membrane [40], reduced promoter activity should not only lengthen the lysis time, as shown in a previous study [50], but should also increase the lysis time stochasticity [51,52].

As shown in Figure 3B, our data showed a negative relationship between the *p*_*R*_*' *activity, and the MLTs, SDs, and CVs. However, the increase of the *p*_*R*_*' *activity had a diminishing influence on both the MLTs, as has been shown previously [50], and the associated SDs and CVs (see Table 2). Interestingly, linear regressions (Figure 3C) showed a much tighter, positive relationship between the MLTs and the SDs (*F*_[1,3]_ = 81.04, *p *= 0.0029; adjusted *R*^*2 *^= 0.952; *y *= -15.7 + 0.3*x*) and a significant positive relationship between the MLTs and CVs (*F*_[1,3]_ = 14.51, *p *= 0.0318, result not shown in the figure). That is, for the WT *S *gene, every 1 minute increase in the MLT corresponds to 0.3 minute increase in lysis time stochasticity.

**Table 2:** Effect of late promoter activity, lysogen growth rate and KCN addition on the stochasticity of lysis time.

Treatment	***n***^**c**^	MLT (min)	SD (min)
*p*_*R*_*' *activity			
IN56 (1)^*a*^	230	65.1	3.24
SYP026 (2)^*a*^	128	61.9	3.20
SYP027 (3)^*a*^	45	62.1	2.91
SYP043 (4)^*a*^	43	74.3	9.22
SYP028 (5)^*a*^	70	110.6	17.83
Growth rate			
100% LB^*b*^	230	65.1	3.24
20% LB	233	59.5	3.86
DM+Glc^*b*^	125	70.3	6.30
DM+Gly^*b*^	78	83.8	9.16
KCN addition			
at 25 min	72	52.1	7.12
at 30 min	67	56.6	6.85
at 32 min	61	54.0	4.74
at 34 min	46	55.7	4.33
at 35 min	161	45.4	1.86
at 45 min	151	50.1	1.83
at 55 min	158	57.6	1.45

^*a *^Numbers in the brackets indicate *p*_*R*_*' *activity ranking with 1 being the highest and 5 being the lowest [50]; IN56 data is from Table 2.

^*b *^100%LB data is from Table 2, strain IN56; DM, Davis minimal salts medium; Glc, glucose; Gly, glycerol.

*C *In some cases, the sample size *n *is the pooled number of cells observed across several days. Detailed information can be found in Table S2 of the addition file 1.

### Effect of Host Growth Rates

In general, cells growing at a faster rate have higher concentrations of various biosynthesis machineries [53]. Since the expression of the phage holin gene is entirely dependent on the host, we hypothesized that a lower host growth rate would lead to a lower rate of holin protein synthesis, thus resulting in a longer lysis time and increased lysis time stochasticity. In the phage T4, it was shown that lysis time was negatively correlated with host growth rate [54].

We determined the MLTs and SDs for wild-type l lysogen grown in four different growth media: standard LB (lysogeny broth [55]), 20% LB, Davis minimal salts medium (DM) with 20 mM glucose, and DM with 40 mM glycerol, resulting in growth rates of 1.01 ± 0.07, 0.93 ± 0.05, 0.49 ± 0.04, and 0.35 ± 0.01 h^-1 ^(mean ± 95% confidence limits), respectively (see Table 2). As shown in Figure 3D, lower growth rates led to increased lysis time SDs (*F*_[1,2]_ = 24.50, *p *= 0.0385) and CVs (*F*_[1,2]_ = 46.24, *p *= 0.0209). A similar negative relationship was also apparent for the MLTs. However, because of the case of the LB medium, in which the higher growth rate actually resulted in a slightly longer MLT, the observed negative relationship was not significant (*F*_[1,2]_ = 6.44, *p *= 0.1265). Interestingly, neither the SDs (*F*_[1,2]_ = 16.11, *p *= 0.0568) nor the CVs (*F*_[1,2]_ = 6.04, *p *= 0.133) was significantly associated with the MLTs.

### Effects of KCN Addition

The energy poison potassium cyanide, KCN, has long been used in phage research to trigger premature lysis [43]. Typically, after KCN addition, culture turbidity declines precipitously [44], indicating that individual lysis events are relatively synchronous. The KCN-induced premature lysis is thought to be mediated through a collapsed proton motive force (PMF) resulting from a inhibition of the bacterial respiratory chain. As has been shown with λ S holin, a 40% drop in the PMF triggers lysis [45]. Without a constant supply of ATP, the production of holin protein would also be terminated. If KCN is added soon after thermal induction of the lysogen culture, few holin proteins would have been made before the termination of holin production. Consequently, it should take a longer time for the holin proteins in the membrane to transition from a diffused state to aggregated rafts. Therefore, after the cessation of holin production by KCN addition, it may take a longer time, on average, before any lysis events are observed. On the other hand, if KCN is added late, a larger proportion of the thermally-induced lysogenic cells should have accumulated enough holin proteins in the cell membrane such that they could be triggered to form holin holes quickly. That is, the addition of KCN should prompt the rapid formation of holin holes, thus resulting in an almost immediate and synchronous lysis of most of the cells in the population. Based on the aforementioned scenarios, we expected that (1) the time delay between the time of KCN addition (*t*_KCN_) and the eventual mean lysis time (*t*_L_) (*i.e.*, *t*_L _- *t*_KCN_) would be negatively correlated with the timing of KCN addition, and (2) *t*_KCN _would be negatively correlated with lysis time stochasticity.

**Figure 4:**
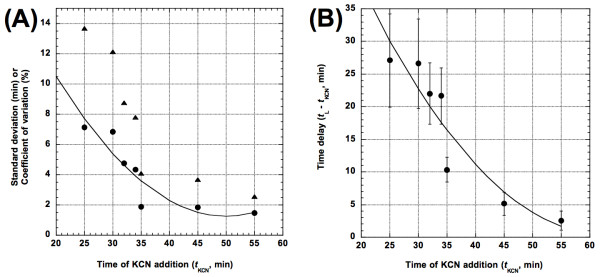
**Effects of *t*_KCN _(timing of KCN addition)**. (A) On time delay *t*_L _- *t*_KCN_. The solid curve shows the quadratic fit of *y *= 54.52 - 1.09*x *+ 0.02(*x *- 36.57)^2^. Error bars indicate the associated SDs. As an example, when *t*_KCN _= 45 min, the observed *t*_L _is 50.11 min, thus the time delay is *t*_L _- *t*_KCN _= 5.11 min. (B) On lysis time SD (closed circles) and CV (closed triangles). Solid curve shows the quadratic fit of SD against *t*_KCN _(*y *= 13.24 - 0.28*x *+ 0.01(*x *- 36.57)^2^).

Figure 4A shows a significant negative relationship between *t*_L _- *t*_KCN _and *t*_KCN_. As KCN was added later in time (*i. e.*, closer to the normal lysis time of 65.1 min), the time delay between addition of KCN and the MLT was reduced (a quadratic fit, *F*_[2,4]_ = 12.87, *p *= 0.0181, adjusted *R*^2 ^= 0.798). In fact, when added 55 min after induction (*i.e.*, 10 min before the normal MLT), the time delay was only 2.6 min, almost instantaneous when compared to the 2 min sampling rate of the sipper-equipped spectrophotometer method of lysis time determination [46]. Interestingly, a theoretical study of lysis time stochasticity by Ryan and Rutenberg also showed a seemingly convex relationship between *t*_L _- *t*_KCN _and *t*_KCN _[[39], their figure five].

The effects of *t*_KCN _on lysis time SDs and CVs are shown in Figure 4B. Again, we witnessed the expected pattern of a significant negative relationship between *t*_KCN _and the SDs (a quadratic fit, *F*_[2,4]_ = 9.91, *p *= 0.0123, adjusted *R*^2 ^= 0.748) and between *t*_KCN _and the CVs (a quadratic fit, *F*_[2,4]_ = 16.03, *p *= 0.0282, adjusted *R*^2 ^= 0.834). These results showed that the later in time KCN was added, the less variation there was in individual lysis times. In fact, the lowest SD (1.45 min) and lowest CV (2.53%) were observed when KCN was added 55 min after induction. This was a significant two-fold reduction in the SD when compared normal lysis conditions (see Table 1 for strain IN56 with the SD = 3.24 min; Student's *t *= 15.45, *p *< 0.0001, using the standard deviation for the SD in Box 7.1 of [56]). This observation indicated that individual triggering for hole formation during the normal progression of cell lysis was relatively asynchronous when compared to the artificial method of acute triggering by KCN addition.

Similar to the effect of growth rate, a linear regression of the SDs (*F*_[1,5]_ = 0.60, *p *= 0.4726) or CVs (*F*_[1,5]_ = 0.328, *p *= 0.5917) against the MLTs did not yield significant result. Another interesting aspect of the relationship between *t*_KCN _and the lysis time SDs is that the SDs drop precipitously when KCN is added about 35 min after induction. This observation suggests that, approximately 35 min after thermal induction, the majority of the lysogenic cells have accumulated enough holin proteins in the cell membrane to form holes immediately if triggered.

## Discussion

The current model of holin hole formation hypothesizes that λ phage lysis timing is mainly determined by when a critical concentration of holin proteins is reached in the cell membrane [40] (Figure 1, dark arrows). According to this model, any factor that influences the holin protein production should also affect the timing of lysis. Furthermore, the realized rate of holin production in each cell should also be subjected to stochastic influences impacting the various upstream biochemical reactions, such as gene transcription and translation, that lead to holin production. As has been shown by others, the lower the average rates of the biochemical reactions, the more prominent the cell-to-cell variation is [51,52].

### Manipulation of holin production rate

In our study, we manipulated the holin production rate by manipulating the λ *p*_*R*_*' *activity and the lysogen growth rate. We observed that, in general, treatments expected to result in higher holin production rates (*e.g.*, high *p*_*R*_*' *activity or high lysogen growth rate) also resulted in shorter MLTs and smaller SDs (Figure 3B and 3D). Furthermore, it was surprising that the combined MLTs and SDs, despite being from two different experimental treatments, namely *p*_*R*_*' *activity and lysogen growth rate, showed almost identical positive correlations, even after excluding the far-flung data point with the longest MLT and largest SD (obtained with strain SYP028, see Table 2) from the analysis (Figure 3C). This result suggests that, irrespective of how the MLT was achieved, as long as the MLTs are the same, we should expect to observe similar SDs. For the wild-type λ S holin sequence, any factor that results in 1.0 min increase in MLT would be accompanied by a concomitant 0.3 min increase in the SD. It would be interesting to conduct a similar experiment with different holin sequences to see if the rate of SD increase is sequence-specific.

Regarding the effects of host growth rate on lysis time stochasticity, it is interesting to note the following. Amir *et al. *[10] found that the MLTs, SDs, and CVs, following UV induction, ranged from 72 min, 9 min, and 12.5% respectively for λ lysogens alone to 99 min, 14 min, and 14.1% respectively for λ lysogens carrying pR-GFP reporter plasmid and 117 min, 19 min, and 15.8% respectively for λ lysogens carrying pR'-tR'-GFP reporter plasmid (all values are extracted from their figures six A and B). Since their λ lysogens were grown in M9 minimal salts medium plus various growth factors and 0.4% glucose at 37°C, it is similar to our Davis minimal salts medium with glucose, from which we obtained the comparable values of 70.3 min, 6.3 min, and 8.96% respectively (see Table 2). It is not clear whether the difference between these two SDs is the result of different methods used for lysogen induction (thermal vs. UV induction) or different growth media, but the MLTs are virtually identical. Their result also indirectly confirmed our current result that host physiology (which is presumably somewhat perturbed in their lysogen strains carrying the medium-copy reporter plasmids) would affect the overall MLTs and SDs of lysis time.

### Manipulation of holin protein sequence

Barring potential post-translational modifications due to differences in holin protein sequence (*e.g.*, differential rate in proteolysis), isogenic λ strains expressing different holin sequences would have a similar average rate of holin accumulation in the membrane and consequently the same distribution of holin proteins among the cells across different lysogen populations. That is, at any given moment, we would expect a certain proportion of cells to accumulate a certain number of holin molecules in the membrane, irrespective of the holin sequences. The observed differences in MLTs, as the result of differences in holin sequence, can be seen as a reflection of different set-points for critical concentrations in an increasingly crowded cell membrane [40]. Presumably, a sequence with a lower set-point would not only result in a shorter MLT, but also a smaller SD as well. However, the existence of similar MLTs, but very different SDs, suggests that missense mutations in the holin sequence not only affect the set-point for spontaneous triggering, but also impact the robustness of the set-point. For example, some mutations may be relatively insensitive to the critical holin concentration, thus resulting in proportionally more cells that are triggered earlier and later than expected, hence greater lysis time stochasticity.

### Effect of energy poison KCN

It is well known that addition of the energy poison, KCN, to induced lysogen cultures will accelerate the onset of lysis [44]. Our results also confirmed this observation (see Table 2). However, it is not clear how this accelerated lysis would affect the lysis time stochasticity. From anecdotal observations, the addition of KCN seems to synchronize lysis, thus resulting in a precipitous decline of lysogen culture turbidity. Our study showed that the timing of KCN addition was inversely related to lysis time stochasticity (see Figure 4B). In fact, the smallest SD (1.45 min) was achieved by adding KCN at 55 min after thermal induction (see Table 2), a time where normally only about 1% of the cells have lysed. The almost synchronous lysis when KCN was added 55 min post thermal induction suggests that most cells would have already accumulated enough holin proteins in the cell membrane to form a hole.

Besides collapsing the PMF, the addition of KCN should also halt the production of holin protein, thus "fixes" the amount of holin proteins on the cell membrane at the time of addition. The progressive decline in lysis time stochasticity as KCN was added later in time (see Figure 4B) strongly suggests that a larger supply of holin protein is a key factor in ensuring synchronous lysis. As more holin proteins are inserted into the cell membrane, the kinetics of raft formation gradually shifts from stochastic to deterministic and synchronous. In fact, there was a nearly five-fold decrease in lysis time stochasticity when the PMF was collapsed at 55 min after lysogen induction when compared to collapse at 25 min (see Table 2). It is also noted that the properties of the normally triggered and the prematurely triggered holin holes are quite distinct, with the prematurely triggered holes being much smaller than the normally triggered holes [28].

### Evolutionary implication of lysis time stochasticity

Both theoretical and experimental studies have demonstrated the importance of lysis timing on phage fitness [46,57-61]. However, it is not clear if lysis time stochasticity would have any impact on phage fitness. All else being equal, genotypes with reduced variances in offspring number would, in the long run, have higher fitness than genotypes that have the same mean offspring number, but larger variances [62,63]. Overall, this suggests that natural selection would tend to minimize stochasticity in phenotypes that are closely linked to Darwinian fitness. If the phage burst size is positively linked with the lysis time, as has been shown previously [46], then selection for reduced burst size stochasticity should lead to reduced lysis time stochasticity as well. Presumably, this hypothesis can be tested by competing two isogenic phage strains that have the same MLTs but very different lysis time SDs. Interestingly, inspection of Table 1 revealed that mutations introduced into WT λ holin sequence usually result in increased stochasticity, except in one case. It is not clear if this observation implies that the WT holin sequences have already been selected for reduced stochasticity in the wild as well. Experiments with more phage holins should provide some hints in this respect.

## Conclusions

Even in a seemingly uniform environment, the lysis time can vary greatly among individual λ lysogenic cells (lysis time stochasticity). The extent of stochasticity, as quantified by the standard deviation, depends on the quality (due to isogenic λ lysogens expressing different S protein alleles) and quantity (manipulated by having different *p*_*R*_*' *activities and lysogen growth rates) of the holin protein, the major determinant of lysis timing in large-genome phages. There is a general positive trend between the mean lysis time and the degree of stochasticity. However, this positive relationship is much tighter when difference in mean lysis time is due to holin quantity rather than quality. The pattern of lysis time stochasticity obtained by addition of KCN at various time points after lysogen induction showed a negative relationship between the timing of KCN addition and the level of lysis time stochasticity.

## Appendix A

This section provides the rationale for partitioning lysis time variance found in the study by Amir *et al. *[10].

For each UV-induced λ lysogenic cell, the lysis time *T *can be divided into three time intervals: (1) *t*_1_, the time interval between lysogen induction and the onset of *p*_*R *_promoter, (2) *t*_2_, the time interval between the onset of the *p*_*R *_promoter and the onset of the *p*_*R*_*' *promoter, and (3) *t*_3_, the time interval between the onset of the *p*_*R*_*' *promoter and the eventual lysis. The following relationships describe the above time intervals and the empirically determined time intervals by Amir *et al. *[10]: *t*_1 _= *t*_pR_, *t*_1 _+ *t*_2 _= *t*_pR'-tR'_, *t*_1 _+ *t*_2 _+ *t*_3 _= *t*_lysis_, and *t*_3 _= Δ*t *= *t*_lysis _- *t*_pR'-tR'_.

For, *T *= *t*_1 _+ *t*_2 _+ *t*_3_, the variance for the lysis time can be expressed as *VAR*(*T*) = *VAR*(*t*_1_) + *VAR*(*t*_2_) + *VAR*(*t*_3_) + 2*COV *(*t*_1_, *t*_2_) + 2*COV *(*t*_2_, *t*_3_) + 2*COV *(*t*_1_, *t*_3_). While the authors did not provide all possible combinations of covariance, it is empirically determined that *COV*(*t*_1 _+ *t*_2_, *t*_3_) = 0, as shown in their figure seven E (*i.e.*, no correlation between *t*_pR'-tR' _and Δ*t*). That is, *COV*(*t*_1 _+ *t*_2_, *t*_3_) = *COV*(*t*_1_, *t*_3_) + *COV*(*t*_2_, *t*_3_) = 0. Although not empirically demonstrated, it seems unlikely that the timing of turning on either the *p*_*R *_or *p*_*R*_*' *promoter would have a positive or negative effect on the assembly of lysis apparatus such that their effects would cancel each other out, resulting in the observed *COV*(*t*_1_, *t*_3_) + *COV*(*t*_2_, *t*_3_) = 0. Most likely, time intervals are mutually independent, *i.e.*, *COV*(*t*_1_, *t*_3_) = *COV*(*t*_2_, *t*_3_) = 0.

The standard deviations ("absolute noise" in their terminology) for *t*_pR'-tR' _and *t*_lysis _can be extracted from their figure six A using data determined from cells carrying the pR'-tR'-GFP plasmid. The estimated SDs for *t*_pR'-tR' _and *t*_lysis _are ~10 min and ~18 min, respectively; therefore, *VAR*(*t*_pR'-tR'_) = ~100 and *VAR*(*t*_lysis_) = ~324. The SD for *t*_pR _can be estimated by extrapolating the line connecting between lysis and *p*_*R*_*' *onset to the 20 min mean time at the *x*-axis (based on the result from cells carrying the pR-GFP plasmid in their figure six A). The corresponding SD for *t*_pR _is ~7 min, thus *VAR*(*t*_pR_) = ~49. Taken together, *VAR*(*t*_1_) = 49, *VAR*(*t*_2_) = 51 (= *VAR*(*t*_1 _+ *t*_2_) - *VAR*(*t*_1_) = 100 - 49 ), and *VAR*(*t*_3_) = 224 (= *VAR*(*t*_1 _+ *t*_2 _+ *t*_3_) - *VAR*(*t*_1 _+ *t*_2_) = 324 - 100). That is, *VAR*(*t*_1_), *VAR*(*t*_2_), and *VAR*(*t*_3_) contributed to 15%, 16%, and 69% of total lysis time variance, respectively.

## Appendix B

Studies of molecular stochasticity typically use the coefficient of variation (CV) as the measurement for the degree of stochasticity [15,25,48,49]. Since CV is a composite statistic (defined as standard deviation/mean), it is sometimes difficult to discern whether an increase in the observed stochasticity (as quantified by CV) is due to decrease in mean or increase in SD. In some cases, a different metric, such as phenotypic noise strength (defined as variance/mean) [17,20], or a slight variant of it (defined as variance/squared mean) [19], has been used as well. Many times, it is not clear why a particular metric is used, except in the instance where the phenotypic noise strength is used to test against an *a priori *expectation of a Poisson distribution, for which variance/mean = 1.

It is understandable why the CV, or a variant, is used in certain situations. For example, if the means are drastically different from each other or a comparison is made between measurements using different units [[56], pp. 57-59.]. In our study, however, the means were not very different and the same measuring unit (*i.e.*, min) was used. Therefore, we presented our means and SDs separately and then jointly as CVs. Except in one instance where presenting stochasticity as SD or CV makes a difference (*i.e.*, effect of genotype on SD or CV vs. MLT), all the other results showed that SD and CV followed the same trend. Since CV can be derived from SD and mean, no information is lost by presenting them separately. Furthermore, when the stochasticity (or noise distribution) is not apparent, it may be advisable to present results as means and SDs (and if necessary, higher moments like skewness and kurtosis) separately, so that the impact of various experimental treatments on the noise distribution can be fully appreciated.

## Methods

### Bacterial strains

All bacteria and phage strains used in this study are listed in Table 3. The copy number of λ genome was checked by PCR following the method of Powell *et al. *[64].

**Table 3:** Bacterial strains used in this study.

Strain	Relevant Genotype^*a*^	Source
IN56	MC4100 (λ *cI857 S*)	[46]
IN57	MC4100 (λ *cI857 S*_*C51S*_)	unpublished strain
IN61	MC4100 (λ *cI857 S105*_*C51S*_)	[46]
IN62	MC4100 (λ *cI857 S105*)	[46]
IN63	MC4100 (λ *cI857 S105*_*C51S/S76C*_)	[46]
IN64	MC4100 (λ *cI857 S*_*C51S/F94C*_)	[46]
IN65	MC4100 (λ *cI857 S105*_*C51S/F94C*_)	unpublished strain
IN66	MC4100 (λ *cI857 S*_*S68C*_)	[46]
IN67	MC4100 (λ *cI857 S105*_*C51S/I13C*_)	[46]
IN68	MC4100 (λ *cI857 S105*_*C51S/L14C*_)	[46]
IN69	MC4100 (λ *cI857 S*_*C51S/L14C*_)	[46]
IN70	MC4100 (λ *cI857 S*_*C51S/F78C*_)	unpublished strain
IN71	MC4100 (λ *cI857 S105*_*C51S/F78C*_)	unpublished strain
IN160	MC4100 (λ *cI857 S*_*A52G *_*Cam*)	unpublished strain
SYP026	MC4100 (λ *cI*857 *p*_*R*_*'*-M2), with *p*_*R*_*' *mutations	[50]
SYP027	MC4100 (λ *cI*857 *p*_*R*_*'*-M1), with *p*_*R*_*' *mutations	[50]
SYP028	MC4100 (λ *cI*857 *p*_*R*_*'*-M5), with *p*_*R*_*' *mutations	[50]
SYP043	MC4100 (λ *cI*857 *p*_*R*_*'*-M4), with *p*_*R*_*' *mutations	[50]

^*a *^*S *denotes wild-type holin gene, when expressed would produce both the S105 holin and S107 antiholin proteins. *S105 *signifies the mutant holin gene with its first codon altered from ATG (Met) to TTG (Leu), thus only produces the S105 holin protein.

### Experimental instrumentation

*E. coli *cells lysogenic for λ phage were induced and observed to lyse in a temperature-controlled perfusion chamber. The experimental apparatus consisted of a 250 mL side-arm (on bottom) medium bottle clamped to an elevated support with tubing leading to an inline heater (SH-27B, Warner Instruments, New Haven, CT) that was controlled by a dual channel heater controller (TC-344B, Warner Instruments, New Haven, CT). The growth medium, flowing at a rate of ~1 mL/min (driven by gravity) and heated by the inline heater to the desired temperature, was introduced to a 358 μL perfusion chamber (RC-21B, Warner Instruments, New Haven, CT) mounted on a heating platform (PM2, Warner Instruments, New Haven, CT) that was controlled by the same dual channel heater controller to maintain the desired temperature. The internal temperature of the perfusion chamber was independently monitored by a thermistor. Waste flowed out of the perfusion chamber, pooled in a reservoir, and was siphoned into a 2 L bottle by a vacuum source. Both the perfusion chamber and the heating platform were placed on the stage of an inverted microscope (TS100, Nikon) for observation at 400× magnification. One of the microscope's ocular lenses was replaced with a 10X MiniVID™ microscope camera (LW Scientific, Norcross, GA) to record individual lysis events onto a laptop computer at the rate of 1 frame per second. All data were collected in unit of seconds, though the results were presented in minutes.

### Sample preparation and lysis time determination

Lysogens were cultured overnight in LB or minimal salts media (see below) at 30°C on a rolling drum. Stationary phase cultures were diluted 100-fold in LB or minimal salts media, then grown to A_550 _~ 0.2. 200 μL of exponentially growing cells were immobilized on a 22 mm square glass coverslip that has been pretreated with 0.01% tissue-culture tested poly-L-lysine (mol. wt. 150 K - 300 K, Sigma, St. Louis, MO) at room temperature for 30 min. After assembling the perfusion chamber, the device was immediately placed on the heating platform and infused with heated medium to maintain the chamber temperature at 30°C for 30 min to stabilize the cells. To induce lysis, the chamber temperature was raised to 42°C for 15 min, and then dropped to 37°C for the duration of the observation period (*i.e.*, until ~95% of cells are lysed). Video recording was initiated at the time when the temperature was raised to 42°C. Under these conditions, it usually takes less than 5 min for the temperature to rise from 30°C to 42°C, a transition comparable to shifting culture flasks from a 30°C to 42°C waterbath shaker. Some experiments were performed by adding KCN to the growth medium in the sidearm feeder bottle to a final concentration of 20 mM.

Videos were subsequently analyzed using Windows Media Player™ playback. The times of individual lysis events were then noted visually and recorded manually. The lysis time was defined as the time from the initiation of the first temperature shift to when the image of the cell disappeared from view. In general, it takes about a few seconds (frames) for lysing cells to fully disappear from view (Figure 1A).

### Determination of lysogen growth rate

Lysogen growth rate was manipulated by using different growth medium formulations: (i) full-strength LB (10 g tryptone, 5 g yeast extract, 10 g NaCl per L dH_2_O), (ii) one-fifth-strength LB (2 g tryptone, 1 g yeast extract, 10 g NaCl per L dH_2_O), (iii) 20 mM glucose in Davis minimal salts (7 g K_2_HPO_4_, 2 g KH_2_PO_4_, 1 g (NH_4_)_2_SO_4_, 0.5 g sodium citrate•2H_2_O, and 0.2 g MgSO_4_•7H_2_O), and (iv) 40 mM glycerol in Davis minimal salts. We assessed the growth of the lysogen strain IN56 by culturing it overnight at 30°C in each growth media. The next day, 90 μL of the overnight culture was used to inoculate 25 mL growth medium and the culture was placed in a 30°C waterbath shaker at 220 rpm. Culture growth was followed with a sipper-equipped spectrophotometer at A_550_. The growth rate was calculated as the slope of the linear regression of natural-logarithm transformed A_550 _values over time.

### Statistical analysis

In most cases, data collection for a given strain or treatment spanned several days. Therefore, even for the same lysogen strain or experimental treatment the means and/or variances may be significantly different among data collected from different dates. We conducted a preliminary exploration of our data set using the software package JMP version 7.0.2, as implemented in MacOS operating system. For each lysogen strain or experimental treatment, the means and standard deviations (SDs) were extracted from the data set according to the date the data were collected and were treated as replicates. Pairwise comparisons of the means (using the Tukey-Kramer HSD test) showed that, for more than half of the cases, at least one mean was significantly different from the others. Since we were mainly interested in the variation, we subsequently converted all values into their corresponding residuals (centered by their corresponding means). We also tested the homogeneity of variance from each date replicate, using O'Brien's test, Brown-Forsythe test, Levene's test, and Bartlett's test, all implemented in JMP. Not surprisingly, more than half of the cases showed that at least one replicate variance was significantly different from the others. Although we did not have an *a priori *expectation of lysis time distribution, we nonetheless tested to see if the lysis time in each replicate is normally distributed or not, using the Shapiro-Wilk W test. Again, in many cases, the replicates do not show a normal distribution. Despite variability in our data set, none of our conclusions were fundamentally changed. Therefore, for the presented results, the mean and standard deviation for each lysogen strain or experimental treatment were calculated based on the following criteria: (*i*) if the means and variances were the same among all blocks, then all the data would be pooled together to estimate the combined means and SDs, (*ii*) if the means were significantly different, but the variances were the same among all blocks, then the mean would be estimated by averaging the block means while the SDs would be estimated by pooled residuals, and (*iii*) if the means and variances were significantly different among all blocks, then the means and SDs would be estimated by averaging block means and SDs. For details of our data set, see additional file 1.

## Competing interests

The authors declare that they have no competing interests.

## Authors' contributions

JJD was responsible for conducting all the relevant experiments, data analyses, and the preparation of the manuscript. INW was responsible for the supervision, data analyses, and preparation of the manuscript. Both authors read and approved the final manuscript.

## Supplementary Material

Additional file 1 **Sample sizes and standard deviations**. More detailed data sets for both Table 1 and Table 2. Click here for file

## References

[B1] Avery SV Microbial cell individuality and the underlying sources of heterogeneity Nat Rev Microbiol 2006 4 577 587 10.1038/nrmicro1460 16845428

[B2] Longo D Hasty J Dynamics of single-cell gene expression Mol Syst Biol 2006 2 64 17130866 10.1038/msb4100110 PMC1682029

[B3] Losick R Desplan C Stochasticity and cell fate Science 2008 320 65 68 10.1126/science.1147888 18388284 PMC2605794

[B4] Rao CV Wolf DM Arkin AP Control, exploitation and tolerance of intracellular noise Nature 2002 420 231 237 10.1038/nature01258 12432408

[B5] Raser JM O'Shea EK Noise in gene expression: origins, consequences, and control Science 2005 309 2010 2013 10.1126/science.1105891 16179466 PMC1360161

[B6] Davidson CJ Surette MG Individuality in bacteria Annu Rev Genet 2008 42 253 268 10.1146/annurev.genet.42.110807.091601 18652543

[B7] Fraser D Kaern M A chance at survival: gene expression noise and phenotypic diversification strategies Mol Microbiol 2009 71 1333 1340 10.1111/j.1365-2958.2009.06605.x 19220745

[B8] McAdams HH Arkin A It's a noisy business! Genetic regulation at the nanomolar scale Trends Genet 1999 15 65 69 10.1016/S0168-9525(98)01659-X 10098409

[B9] Veening JW Smits WK Kuipers OP Bistability, epigenetics, and bet-hedging in bacteria Annu Rev Microbiol 2008 62 193 210 10.1146/annurev.micro.62.081307.163002 18537474

[B10] Amir A Kobiler O Rokney A Oppenheim AB Stavans J Noise in timing and precision of gene activities in a genetic cascade Mol Syst Biol 2007 3 71 17299413 10.1038/msb4100113 PMC1828745

[B11] Arkin A Ross J McAdams HH Stochastic kinetic analysis of developmental pathway bifurcation in phage λ-infected *Escherichia coli *cells Genetics 1998 149 1633 1648 9691025 10.1093/genetics/149.4.1633 PMC1460268

[B12] Pearl S Gabay C Kishony R Oppenheim A Balaban NQ Nongenetic individuality in the host-phage interaction PLoS Biol 2008 6 e120 10.1371/journal.pbio.0060120 18494559 PMC2386839

[B13] St-Pierre F Endy D Determination of cell fate selection during phage lambda infection Proc Natl Acad Sci USA 2008 105 20705 20710 10.1073/pnas.0808831105 19098103 PMC2605630

[B14] Cai L Friedman N Xie XS Stochastic protein expression in individual cells at the single molecule level Nature 2006 440 358 362 10.1038/nature04599 16541077

[B15] Elowitz MB Levine AJ Siggia ED Swain PS Stochastic gene expression in a single cell Science 2002 297 1183 1186 10.1126/science.1070919 12183631

[B16] Ito Y Toyota H Kaneko K Yomo T How selection affects phenotypic fluctuation Mol Syst Biol 2009 5 264 19401676 10.1038/msb.2009.23 PMC2683726

[B17] Ozbudak EM Thattai M Kurtser I Grossman AD van Oudenaarden A Regulation of noise in the expression of a single gene Nat Genet 2002 31 69 73 10.1038/ng869 11967532

[B18] Maamar H Raj A Dubnau D Noise in gene expression determines cell fate in *Bacillus subtilis* Science 2007 317 526 529 10.1126/science.1140818 17569828 PMC3828679

[B19] Bar-Even A Paulsson J Maheshri N Carmi M O'Shea E Pilpel Y Barkai N Noise in protein expression scales with natural protein abundance Nat Genet 2006 38 636 643 10.1038/ng1807 16715097

[B20] Blake WJ M KA Cantor CR Collins JJ Noise in eukaryotic gene expression Nature 2003 422 633 637 10.1038/nature01546 12687005

[B21] Fraser HB Hirsh AE Giaever G Kumm J Eisen MB Noise minimization in eukaryotic gene expression PLoS Biol 2004 2 e137 10.1371/journal.pbio.0020137 15124029 PMC400249

[B22] Acar M Mettetal JT van Oudenaarden A Stochastic switching as a survival strategy in fluctuating environments Nat Genet 2008 40 471 475 10.1038/ng.110 18362885

[B23] Ansel J Bottin H Rodriguez-Beltran C Damon C Nagarajan M Fehrmann S Francois J Yvert G Cell-to-cell stochastic variation in gene expression is a complex genetic trait PLoS Genet 2008 4 e1000049 10.1371/journal.pgen.1000049 18404214 PMC2289839

[B24] Blake WJ Balazsi G Kohanski MA Isaacs FJ Murphy KF Kuang Y Cantor CR Walt DR Collins JJ Phenotypic consequences of promoter-mediated transcriptional noise Mol Cell 2006 24 853 865 10.1016/j.molcel.2006.11.003 17189188

[B25] Bishop AL Rab FA Sumner ER Avery SV Phenotypic heterogeneity can enhance rare-cell survival in 'stress-sensitive' yeast populations Mol Microbiol 2007 63 507 520 17176259 10.1111/j.1365-2958.2006.05504.x

[B26] Wang IN Smith DL Young R HOLINS: The Protein Clocks of Bacteriophage Infections Annu Rev Microbiol 2000 54 799 825 10.1146/annurev.micro.54.1.799 11018145

[B27] Young R Wang IN Roof WD Phages will out: strategies of host cell lysis Trends Microbiol 2000 8 120 128 10.1016/S0966-842X(00)01705-4 10707065

[B28] Wang IN Deaton J Young R Sizing the holin lesion with an endolysin-β-galactosidase fusion J Bacteriol 2003 185 779 787 10.1128/JB.185.3.779-787.2003 12533453 PMC142811

[B29] Savva CG Dewey JS Deaton J White RL Struck DK Holzenburg A Young R The holin of bacteriophage lambda forms rings with large diameter Mol Microbiol 2008 69 784 793 10.1111/j.1365-2958.2008.06298.x 18788120 PMC6005192

[B30] Park T Struck DK Dankenbring CA Young R The pinholin of lambdoid phage 21: control of lysis by membrane depolarization J Bacteriol 2007 189 9135 9139 10.1128/JB.00847-07 17827300 PMC2168629

[B31] Xu M Arulandu A Struck DK Swanson S Sacchettini JC Young R Disulfide isomerization after membrane release of its SAR domain activates P1 lysozyme Science 2005 307 113 117 10.1126/science.1105143 15637279

[B32] Xu M Struck DK Deaton J Wang IN Young R A signal-arrest-release sequence mediates export and control of the phage P1 endolysin Proc Natl Acad Sci USA 2004 101 6415 6420 10.1073/pnas.0400957101 15090650 PMC404059

[B33] Zhang N Young R Complementation and characterization of the nested *Rz *and *Rz1 *reading frames in the genome of bacteriophage lambda Mol Gen Genet 1999 262 659 667 10.1007/s004380051128 10628848

[B34] Berry J Summer EJ Struck DK Young R The final step in the phage infection cycle: the Rz and Rz1 lysis proteins link the inner and outer membranes Mol Microbiol 2008 70 341 351 10.1111/j.1365-2958.2008.06408.x 18713319 PMC4623567

[B35] Young R Way J Way S Yin J Syvanen M Transposition mutagenesis of bacteriophage lambda: a new gene affecting cell lysis J Mol Biol 1979 132 307 322 10.1016/0022-2836(79)90262-6 160463

[B36] Friedman DI Gottesman M Hendrix RW,Roberts JW,Stahl FW,Weisberg RA Lytic mode of lambda development Lambda II 1983 Cold Spring Harbor, New York: Cold Spring Harbor Laboratory 21 51

[B37] Gründling A Bläsi U Young R Genetic and biochemical analysis of dimer and oligomer interactions of the lambda S holin J Bacteriol 2000 182 6082 6090 10.1128/JB.182.21.6082-6090.2000 11029428 PMC94742

[B38] Dewey JS Savva CG White RL Vitha S Holzenburg A Young R Micron-scale holes terminate the phage infection cycle Proc Natl Acad Sci USA 2010 107 2219 2223 10.1073/pnas.0914030107 20080651 PMC2836697

[B39] Ryan GL Rutenberg AD Clocking out: modeling phage-induced lysis of *Escherichia coli* J Bacteriol 2007 189 4749 4755 10.1128/JB.00392-07 17468251 PMC1913442

[B40] White R Chiba S Pang T Dewey JS Savva CG Holzenburg A Pogliano K Young R Holin triggering in real time Proc Natl Acad Sci USA 2010 108 798 803 21187415 10.1073/pnas.1011921108 PMC3021014

[B41] Ellis EL Delbrück M The growth of bacteriophage J Gen Physiol 1939 22 365 384 10.1085/jgp.22.3.365 19873108 PMC2141994

[B42] Delbrück M The growth of bacteriophage and lysis of the host J Gen Physiol 1940 23 643 660 10.1085/jgp.23.5.643 19873180 PMC2237944

[B43] Doermann AH The intracellular growth of bacteriophages. I. Liberation of intracellular bacteriophage T4 by premature lysis with another phage or with cyanide J Gen Physiol 1952 35 645 656 10.1085/jgp.35.4.645 14898042 PMC2147306

[B44] Young R Bacteriophage lysis: mechanism and regulation Microbiol Rev 1992 56 430 481 1406491 10.1128/mr.56.3.430-481.1992 PMC372879

[B45] Gründling A Manson MD Young R Holins kill without warning Proc Natl Acad Sci USA 2001 98 9348 9352 10.1073/pnas.151247598 11459934 PMC55423

[B46] Wang IN Lysis timing and bacteriophage fitness Genetics 2006 172 17 26 16219778 10.1534/genetics.105.045922 PMC1456144

[B47] Raab R Neal G Garrett J Grimaila R Fusselman R Young R Mutational analysis of bacteriophage lambda lysis gene S J Bacteriol 1986 167 1035 1042 2943725 10.1128/jb.167.3.1035-1042.1986 PMC215977

[B48] Swain PS Elowitz MB Siggia ED Intrinsic and extrinsic contributions to stochasticity in gene expression Proc Natl Acad Sci USA 2002 99 12795 12800 10.1073/pnas.162041399 12237400 PMC130539

[B49] Raj A Peskin CS Tranchina D Vargas DY Tyagi S Stochastic mRNA synthesis in mammalian cells PLoS Biol 2006 4 1707 1719 10.1371/journal.pbio.0040309 PMC1563489 17048983

[B50] Shao Y Wang IN Effect of late promoter activity on bacteriophage λ fitness Genetics 2009 181 1467 1475 10.1534/genetics.108.098624 19171945 PMC2666513

[B51] Gillespie DT Exact stochastic simulation of coupled chemical reactions J Phys Chem 1977 81 2340 2361 10.1021/j100540a008

[B52] McAdams HH Arkin A Stochastic mechanisms in gene expression Proc Natl Acad Sci USA 1997 94 814 819 10.1073/pnas.94.3.814 9023339 PMC19596

[B53] Bremer H Dennis PP Ingraham JL,Low KB,Magasanik B,Schaechter M,Umbarger HE Modulation of chemical composition and other parameters of the cell by growth rate Escherichia coli and Salmonella typhimurium Cellular and Molecular Biology 1987 2 Washington, D.C.: American Society for Microbiology 1527 1542

[B54] Hadas H Einav M Fishov I Zaritsky A Bacteriophage T4 development depends on the physiology of its host *Escherichia coli* Microbiology 1997 143 179 185 10.1099/00221287-143-1-179 9025292

[B55] Bertani G Lysogeny at mid-twentieth century: P1, P2, and other experimental systems J Bacteriol 2004 186 595 600 10.1128/JB.186.3.595-600.2004 14729683 PMC321500

[B56] Sokal RR Rohlf FJ Biometry 1995 3 New York, New York: W. H. Freeman and Company

[B57] Abedon ST Selection for bacteriophage latent period length by bacterial density: A theoretical examination Microb Ecol 1989 18 79 88 10.1007/BF02030117 24196124

[B58] Abedon ST Herschler TD Stopar D Bacteriophage latent-period evolution as a response to resource availability Appl Environ Microbiol 2001 67 4233 4241 10.1128/AEM.67.9.4233-4241.2001 11526028 PMC93152

[B59] Heineman RH Bull JJ Testing optimality with experimental evolution: lysis time in a bacteriophage Evolution 2007 61 1695 1709 10.1111/j.1558-5646.2007.00132.x 17598749 PMC1974807

[B60] Shao Y Wang IN Bacteriophage adsorption rate and optimal lysis time Genetics 2008 180 471 482 10.1534/genetics.108.090100 18757924 PMC2535697

[B61] Wang IN Dykhuizen DE Slobodkin LB The evolution of phage lysis timing Evol Ecol 1996 10 545 558 10.1007/BF01237884

[B62] Gillespie JH Nautural selection for within-generation variance in offspring number Genetics 1974 76 601 606 4833578 10.1093/genetics/76.3.601 PMC1213089

[B63] Gillespie JH Natural selection for variances in offspring numbers: a new evolutionary principle Am Nat 1977 111 1010 1014 10.1086/283230

[B64] Powell BS Rivas MP Court DL Nakamura Y Turnbough CL Jr Rapid confirmation of single copy lambda prophage integration by PCR Nucleic Acids Res 1994 22 5765 5766 10.1093/nar/22.25.5765 7838735 PMC310146

