# Supplementary material for: Factors influencing lysis time stochasticity in bacteriophage λ
Source: BMC Microbiol. 2011 Aug 2;11:174. doi: 10.1186/1471-2180-11-174 (PMC3166277; doi:10.1186/1471-2180-11-174)
Supplement: Additional file 1 — Sample sizes and standard deviations. More detailed data sets for both Table 1 and Table 2. [file 1471-2180-11-174-S1.DOC]

**Addition files**

**Sample sizes and standard deviations**

| Table S1. Effects of holin sequences on the stochasticity of lysis time. | | | |
| --- | --- | --- | --- |
| Strain | *n* | MLT (min) | SD (min) |
| IN61 | 109 | 44.2 | 2.98 |
|  | 21 | 46.4 | 2.12 |
|  | 72 | 46.3 | 2.78 |
|  | 72 | 46.0 | 3.80 |
| IN56 (WT) | 157 | 64.8 | 4.33 |
|  | 20 | 66.0 | 2.80 |
|  | 53 | 64.5 | 2.60 |
| IN160 | 47 | 29.5 | 3.28 |
| IN62 | 35 | 54.3 | 3.40 |
|  | 59 | 54.5 | 3.72 |
|  | 42 | 54.1 | 3.05 |
| IN70 | 52 | 54.5 | 3.86 |
| IN57 | 53 | 47.0 | 4.25 |
| IN69 | 119 | 45.0 | 4.38 |
| IN63 | 40 | 41.7 | 5.54 |
|  | 49 | 42.4 | 4.39 |
|  | 79 | 38.9 | 3.66 |
|  | 41 | 41.7 | 4.62 |
| IN64 | 63 | 48.4 | 4.60 |
| IN68 | 43 | 56.0 | 7.21 |
|  | 110 | 52.1 | 3.07 |
| IN66 | 25 | 87.0 | 6.50 |
|  | 140 | 75.2 | 3.39 |
|  | 24 | 84.5 | 7.72 |
| IN67 | 46 | 48.3 | 4.83 |
|  | 67 | 55.0 | 4.37 |
|  | 99 | 69.5 | 10.94 |
| IN65 | 33 | 83.8 | 6.95 |
| IN71 | 49 | 68.8 | 7.67 |

| Table S2. Effect of late promoter activity, lysogen growth rate and KCN addition on the stochasticity of lysis time. | | | |
| --- | --- | --- | --- |
| Treatment | *n* | MLT (min) | SD (min) |
| *p_R_'* activity | | | |
| IN56 | see Table S1 |  |  |
| SYP026 | 128 | 61.9 | 3.20 |
| SYP027 | 45 | 62.1 | 2.91 |
| SYP043 | 14 | 75.9 | 4.61 |
|  | 29 | 72.6 | 2.13 |
| SYP028 | 53 | 109.7 | 17.0 |
|  | 17 | 113.3 | 21.0 |
| Growth rate | | | |
| 100% LB | the same as IN56 | | |
| 20% LB | 98 | 59.1 | 3.99 |
|  | 135 | 59.7 | 3.77 |
| DM+Glc | 68 | 78.4 | 5.88 |
|  | 57 | 62.2 | 6.73 |
| DM+Gly | 78 | 83.8 | 9.16 |
| KCN addition | | | |
| at 25 min | 72 | 52.1 | 7.12 |
| at 30 min | 67 | 56.6 | 6.85 |
| at 32 min | 61 | 54.0 | 4.74 |
| at 34 min | 46 | 55.7 | 4.33 |
| at 35 min | 95 | 44.7 | 1.93 |
|  | 66 | 46.0 | 1.76 |
| at 45 min | 79 | 48.8 | 1.67 |
|  | 72 | 51.4 | 2.01 |
| at 55 min | 127 | 57.0 | 1.73 |
|  | 31 | 58.1 | 1.18 |
